# Multidisciplinary Therapeutic Approach of a Patient With Sjogren’s Syndrome: A Three-Year Follow-Up Study

**DOI:** 10.7759/cureus.55148

**Published:** 2024-02-28

**Authors:** Maria Kokoti, Vasileios Zisis, Dimitrios Andreadis, Athina Bakopoulou

**Affiliations:** 1 Prosthodontics, School of Dentistry, Faculty of Health Sciences, Aristotle University of Thessaloniki, Thessaloniki, GRC; 2 Oral Medicine/Pathology, School of Dentistry, Faculty of Health Sciences, Aristotle University of Thessaloniki, Thessaloniki, GRC

**Keywords:** sjogren’s syndrome, oral condition, fixed prosthodontics, prosthetic rehabilitation, fixed partial dentures

## Abstract

Sjögren's syndrome is a chronic, inflammatory autoimmune disorder characterized by lymphocyte infiltration of the exocrine glands. Notably, the rehabilitation of partially edentulous patients with Sjögren's syndrome is limited by the scarce availability of studies that could inform therapeutic modalities and potential challenges during clinical procedures. This case report aimed to present the oral rehabilitation of a patient with Sjögren's syndrome who received fixed partial dentures (FPDs). A 28-year-old female patient sought treatment to restore her missing teeth. She was diagnosed with Sjögren's syndrome by a rheumatologist adhering to the revised version of the European criteria proposed by the American-European Consensus Group and was on a medication regimen including prednisolone, hydroxychloroquine, pantoprazole, pilocarpine, and tear substitutes to manage her condition. The final treatment plan consisted of extractions, management of gingivitis, post-and-core restorations, and a 2 mm vertical dimension increase with the placement of 15 porcelain-fused-to-metal (PFM) crowns and 4 short-span bridges. The patient underwent regular clinical and radiographic evaluations every 3 months since June 2020. Throughout this period, the fixed prostheses, teeth, and periodontal tissues demonstrated remarkable stability and exhibited no complications. This three-year case study provides evidence that meticulous planning and clinical execution can facilitate successful oral rehabilitation in young edentulous patients with Sjögren's syndrome. Tooth-supported fixed prostheses can effectively restore oral function and aesthetic appeal in these individuals, provided they undergo more frequent dental examinations than the general population and maintain a cooperative attitude throughout the treatment process.

## Introduction

Sjögren's syndrome is a chronic, inflammatory, autoimmune disorder characterized by infiltration of the exocrine glands with lymphocytes. The primary symptoms, which manifest in the lacrimal and salivary glands, are dry eyes (xerophthalmia) and dry mouth (xerostomia) [1]. It ranks as the second most prevalent autoimmune disorder after rheumatoid arthritis, with an estimated prevalence of 0.5% to 3% in the general population. Women are disproportionately affected, with a male-to-female ratio of 1:9. The average age of onset is between 40 and 50 years, but Sjögren's syndrome can also occur in children and young adults [1]. For a differential diagnosis of the syndrome, five groups of diagnostic criteria have been suggested: a. Copenhagen’s [2], b. Japanese [3], c. Greek [4], d. California’s - Californian-Fox CF [5], and e. revised version of the European criteria proposed by the American-European Consensus Group [6-8]. A well-rounded treatment strategy should directly tackle the systemic symptoms, carefully adjusting the dosage of medications such as non-steroidal anti-inflammatory drugs (NSAIDs), corticosteroids, immunoregulators, immunodepressors, etc, based on individual needs (symptoms presentation and severity). To alleviate the symptoms of xerostomia and xerophthalmia, a combination of bromhexine, cevimeline, or pilocarpine can be administered, alongside artificial saliva, fluoride, and tear substitutes [9]. The rehabilitation of partially edentulous patients with Sjögren's syndrome is a challenging task, and there is a paucity of studies to guide the selection of appropriate treatment modalities and address potential clinical complications [10]. This case report aimed to present the treatment approach of a patient with Sjögren's syndrome, who received fixed partial dentures.

## Case presentation

A 28-year-old female patient presented to the Department of Prosthodontics, School of Dentistry, Aristotle University of Thessaloniki, Greece, seeking replacement of her missing teeth. She reported a complex medical history, including iron deficiency anemia, lowered hematocrit, hypothyroidism, duodenal ulcer, gastroesophageal reflux disease (GERD), xerophthalmia, “sandy” and “gritty” feeling in her eyes, photosensitivity, xerostomia, difficulty chewing, swallowing, and speaking, and angular cheilitis. Further evaluation by an ophthalmologist and rheumatologist led to a diagnosis of primary Sjögren's syndrome (SS) according to the revised version of the European criteria proposed by the American-European Consensus Group (Table 1) [1,7,11].

**Table 1:** Objective findings antinuclear antibody (ANA); anti-Sjögren's syndrome type B (SSB) antibody; rheumatoid factor (RF)

TEST	RESULT
Schirmer test	positive
Rose Bengal test	positive
Sialometry	positive
Lip biopsy	positive
ANA, SSB, RF	positive
Other rheumatic disease	negative

**Figure 1:**
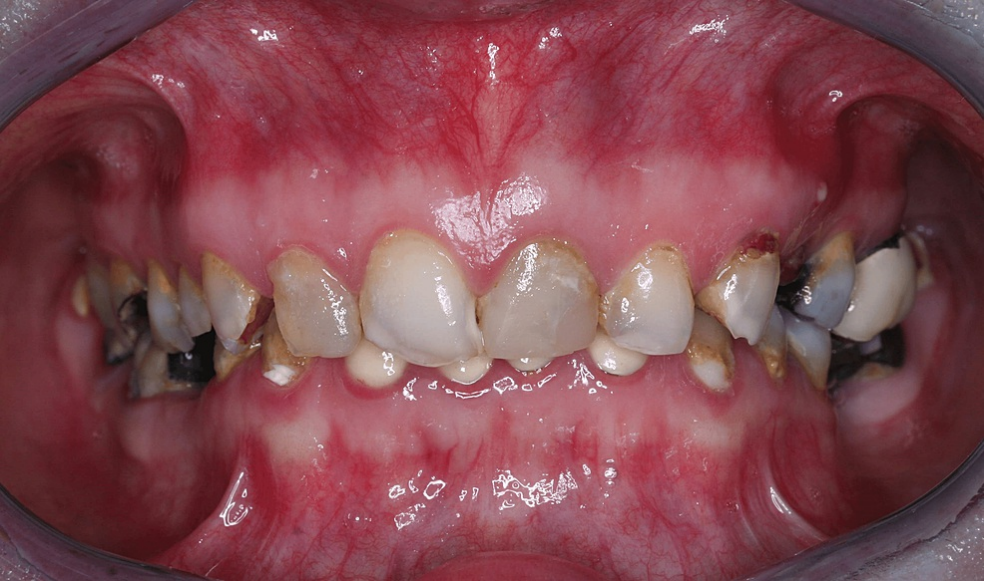
Pre-treatment - interocclusal position

**Figure 2:**
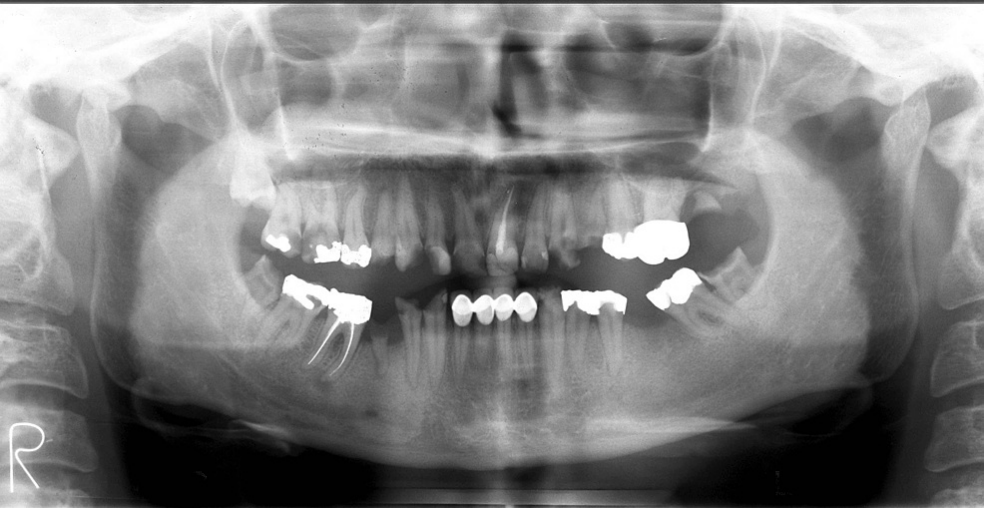
Pre-treatment - panoramic X-ray

The patient is currently under medical management with prednisolone, hydroxychloroquine sulfate, pantoprazole, pilocarpine, and levothyroxine sodium. She also utilizes artificial tears in the form of ophthalmic drops to alleviate her xerophthalmia symptoms. A thorough clinical and radiographic examination of the patient's teeth revealed one missing tooth (#36), remaining roots of teeth #24, 27, and 45, PFM crowns on teeth #26, 31, 32, 41, and 42, periapical lesions regarding teeth #16, 24, and 46, extensive restorations of either amalgam or composite material on the natural teeth, and extensive dental caries and erosions. The patient reported that the dental wear had occurred during the last three years, despite maintaining good oral hygiene habits (Figures 1, 2).

The periodontal evaluation revealed a plaque index (PI) of 0.9 and a gingival index (GI) of 1.

Probing depth measurements are shown in Tables 2, 3.

**Table 2:** Maxillary periodontal measurements

	BUCCALLY															
TOOTH	18	17	16	15	14	13	12	11	21	22	23	24	25	26	27	28
DEPTH (mm)		212	212	213	212	212	312	112	211	212	212		212	112		

	PALATALLY															
TOOTH	18	17	16	15	14	13	12	11	21	22	23	24	25	26	27	28
DEPTH (mm)		212	222	222	212	222	212	111	111	222	222		212	211		

**Table 3:** Mandibular periodontal measurements

	BUCCALLY															
TOOTH	48	47	46	45	44	43	42	41	31	32	33	34	35	36	37	38
DEPTH (mm)	212	212	223		112	222	222	112	211	212	112	212	212		212	212

	LINGUALLY															
TOOTH	48	47	46	45	44	43	42	41	31	32	33	34	35	36	37	38
DEPTH (mm)	222	212	221		212	221	222	112	122	212	212	322	211		111	123

The dental occlusion analysis revealed angle class II, 5 mm overjet, 7 mm overbite, type of occlusion: group function, freeway space: 2 mm, decrease in the vertical dimension, and no symptoms were reported regarding the temporomandibular joint.

Treatment plan

Preoperative impressions and interocclusal records were obtained for treatment planning purposes. These were followed by mounting the study casts on an articulator and creating a diagnostic wax-up. Maxillary and mandibular mock-ups were fabricated for aesthetic evaluation and phonetic trials. Provisional restorations were fabricated replicating the diagnostic wax-up. The final treatment plan encompassed the following procedures: extraction of the teeth #16, 24, 27, 38, and 48, endodontic treatment of the teeth #12, 13, 14, 17, 23, 37, 43, 44, 46, and 47, conservative treatment of the gingivitis, construction of post-and-core restorations for the teeth #12, 13, 14, 17, 21, 23, 37, 43, 44, 46, and 47, two-millimeter increase in vertical dimension followed by fabrication of PFM crowns on teeth #11, 12, 13, 14, 21, 22, 26, 31, 32, 33, 34, 41, 42, 43, and 47, fabrication of PFM bridges on the following segments: #15-17, #23-25, #35-37, and #44-46, and finally fabrication of the night guard. The patient provided informed consent for the publication of her photos.

Oral hygiene instructions and follow-up

**Figure 3:**
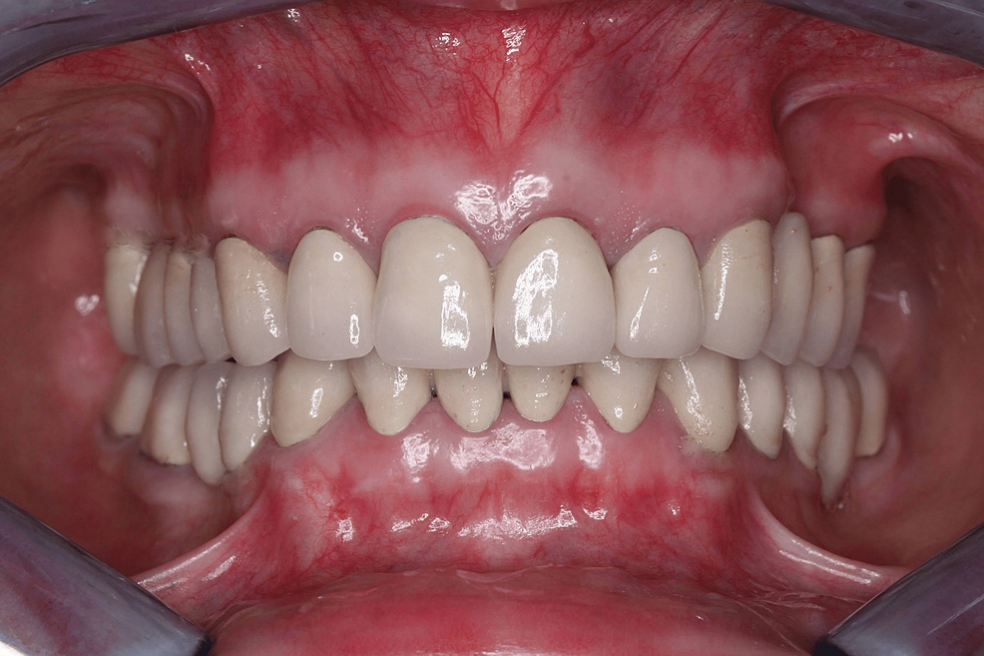
Post-treatment – interocclusal position

**Figure 4:**
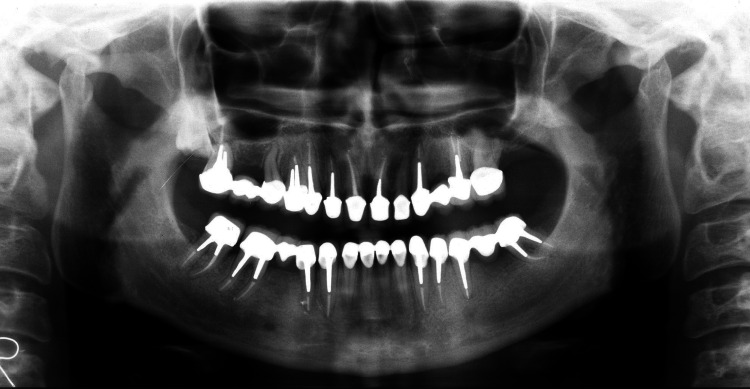
Post-treatment - panoramic X-ray

The patient received oral hygiene instructions tailored to manage xerostomia. These included using a gentle toothpaste and a sugarless chewing gum [8] to stimulate saliva production. She was advised to avoid salty, spicy foods and substances that exacerbate xerostomia, such as caffeine [6], and to increase her fluid intake, particularly water [12]. In addition, the patient was advised to use chlorhexidine mouthwash after brushing [13], to eliminate the proliferation of bacteria and to apply fluoride gel to her teeth daily, using custom-made soft trays [14], to decrease the risk of recurrent caries and prevent further damage [6,15]. Finally, she was scheduled for regular dental checkups every three months, including clinical and radiographic examinations [9,16]. The patient has been compliant with this schedule and has been examined every three months since June 2020. During this time, her teeth and periodontal tissues have remained healthy, and the fixed prostheses have remained stable without any complications (Figures 3, 4).

## Discussion

Our patient's initial signs and symptoms emerged around the age of 25. While it's uncommon for the syndrome to manifest at this age, it's important to consider that the first clinical symptom may not appear for 8 to 10 years [1]. In this case, osseointegrated dental implants were not considered as the primary treatment option. Instead, the patient opted for fixed partial dentures (FPDs) over teeth-supported prostheses [17,18]. This decision was based on several factors, including the patient's age, the presence of numerous natural teeth, financial considerations, the patient's excellent cooperation, and her commitment to maintaining good oral hygiene. Additionally, the patient's high corticosteroid intake was a contraindication for implant placement [19]. While implant-retained prostheses have been shown to improve the function and comfort of fully edentulous patients with Sjögren's syndrome [9,10,19-22], there have been reports of a higher rate of implants not achieving osseointegration or losing osseointegration within the first year following prosthetic rehabilitation [17,23]. Several studies have shown promising results for dental implants in patients with Sjögren's syndrome. These studies report high implant survival rates, ranging from 97% [24] to 100% [25], for complete-arch implant-supported restorations. However, it's important to note that the longest follow-up period in these studies was 36-57.6 months [10]. Albrecht et al. investigated a group of 32 patients with Sjögren's syndrome who received implants. They found that 4.8% of implants had to be removed [26]. The most common challenge faced by patients with Sjögren's syndrome is the premature loss of a significant number of natural teeth due to rampant dental caries brought on by insufficient saliva production [27]. The extensive dental wear and erosions observed in these patients are likely attributed to the lack of salivary protection in the upper gastrointestinal tract [12] and alterations in salivary composition [28]. To minimize the risk of recurrent caries, it was determined that the cervical margins of the restorations should be placed subgingivally to thoroughly protect the underlying natural teeth. Additionally, only full-coverage single crowns and three-unit bridges were fabricated to facilitate straightforward replacement in the event of unforeseen complications [15]. The patient wore provisional restorations for a period of three months, allowing her to acclimate to the increased vertical dimension while simultaneously undergoing a comprehensive evaluation of temporomandibular joint functionality. The clinical procedure presented several challenges that were both difficult for the dentist to manage and uncomfortable for the patient throughout the treatment. These challenges stemmed from the patient's fragile oral tissues, dry and sensitive mucosa, dry, chapped lips, and angular cheilitis. These conditions resulted in significant patient discomfort and intolerance [29]. Despite the absence of active periodontal disease, the patient experienced a persistent tendency for bleeding during procedures [30,31], particularly during tooth preparation and final impressions. This persistent bleeding was likely due to alterations in salivary quantity and quality, particularly pH and buffer capacity [32], but may have also been exacerbated by the medication, which could have contributed to the increased bleeding tendency, edema, vascular dilation, and low platelet count. The aforementioned challenges were addressed carefully through local vasoconstriction, a meticulous focus on achieving maximum marginal accuracy within the boundaries of the provisional and final restorations, and the creation of smooth, even surfaces on both the margins and external surfaces of the restorations. Saliva substitute was not provided to the patient due to its high cost [6], and citrus candies were also withheld due to their potential demineralization effect [8].

## Conclusions

This three-year follow-up study demonstrated that with meticulous planning and execution of the clinical procedure, young edentulous patients with Sjögren's syndrome can undergo FPDs with success, provided they receive more frequent dental examinations than the general population and maintain their cooperation. Therefore, a prompt diagnosis, comprehensive interdisciplinary treatment, regular dental checkups, and effective oral hygiene practices can help patients with Sjögren’s syndrome achieve a satisfactory level of oral health, reduce the frequency of dental issues, and enhance their overall quality of life.
